# Type of Extender and Equilibration Time as Factors Affecting Post-Thaw Quality Characteristics of Turkey Semen

**DOI:** 10.3390/ani15213218

**Published:** 2025-11-05

**Authors:** Sara Ataei-Nazari, Filip Benko, Tomáš Slanina, Jakub Vozaf, Michal Ďuračka, Tomáš Války, Peter Chrenek, Eva Tvrdá, Miroslava Kačániová

**Affiliations:** 1 Institute of Biotechnology, Slovak University of Agriculture in Nitra, Tr. A. Hlinku 2, 949 76 Nitra, Slovakia; sara.ataeinazari@uniag.sk (S.A.-N.); filip.benko@uniag.sk (F.B.); jakub.vozaf@uniag.sk (J.V.); peter.chrenek@uniag.sk (P.C.); evina.tvrda@gmail.com (E.T.); 2 Institute of Applied Biology, Slovak University of Agriculture in Nitra, Tr. A. Hlinku 2, 949 76 Nitra, Slovakia; tomas.slanina@uniag.sk; 3 National Agricultural and Food Center (NPPC), Research Institute for Animal Production Nitra, Hlohovecká 2, 951 41 Lužianky, Slovakia; 4 AgroBioTech Reseach Centre, Slovak University of Agriculture in Nitra, Tr. A. Hlinku 2, 949 76 Nitra, Slovakia; michal.duracka@uniag.sk; 5 Faculty of Science, Masaryk University, Kotlářská 267/2, 611 37 Brno, Czech Republic; thomas.valky@gmail.com; 6 Chair of Animal Breeding and Biotechnology, Estonian University of Life Sciences, Kreutzwaldi 62, 510 06 Tartu, Estonia; 7 Institute of Horticulture, Faculty of Horticulture and Landscape Engineering, Slovak University of Agriculture, Tr. A. Hlinku 2, 949 76 Nitra, Slovakia; 8 School of Medical and Health Sciences, VIZJA University, Okopowa 59, 010 43 Warsaw, Poland

**Keywords:** turkey semen, cryopreservation, semen extender, equilibration time, sperm quality

## Abstract

Commercial turkey breeding relies almost entirely on artificial insemination, yet avian sperm are unusually vulnerable to cooling and freezing injury. As a result, extender chemistry and processing steps, especially low-temperature equilibration, are pivotal for post-thaw performance. We evaluated how extender choice, paired with equilibration time, shapes the post-thaw quality of turkey semen. Ejaculates were diluted in Beltsville, Sperm Motility Medium (SMM), Botucrio, or Kobidil^+^, then equilibrated for 20 or 40 min before freezing; samples were cryostored for one month and assessed immediately after thawing. The outcomes included motility/kinematics, membrane integrity, mitochondrial activity and membrane potential, apoptosis/necrosis, reactive oxygen species (ROS), DNA fragmentation, and bacteriological load. Overall, 20 min equilibration improved post-thaw motility and membrane integrity, and reduced DNA fragmentation and ROS. Among extenders, Beltsville delivered the best overall sperm quality. Considering the extender × time interaction, Beltsville, Botucrio, and Kobidil^+^ performed best at 20 min, whereas SMM performed best at 40 min. Thus, Beltsville and SMM provide strong, time-specific options for turkey semen cryopreservation—Beltsville at 20 min and SMM at 40 min.

## Simple Summary

Avian spermatozoa are highly susceptible to ice-crystal injury, making semen cryopreservation particularly challenging. Post-thaw quality is strongly influenced by the cooling program, equilibration time, extender composition, and choice of cryoprotectants, antioxidants, and antibiotics. Ongoing work therefore seeks species- and even breed-specific optimization of protocols and extenders. In this study, we tested multiple extenders paired with two equilibration times to assess their effects on post-thaw turkey semen quality, revealing that extender performance varied and was dependent on equilibration time.

## 1. Introduction

Extensive selection for breast muscle volume in turkeys—a market-preferred trait—has led to the loss of natural mating ability in males [1]. Consequently, unlike wild turkeys, commercial breeding programs depend almost entirely on artificial insemination, typically using fresh semen since it outperforms cryopreserved semen in motility and fertility endpoints. Nonetheless, cryopreserved semen remains essential for safeguarding elite sires, accelerating genetic gain, and controlling inbreeding, despite the well-recognized consequences of cryotrauma, osmotic shock, and oxidative stress [2,3].

Avian spermatozoa are particularly vulnerable to cooling and freezing compared with mammalian sperm, owing to differences in morphology and membrane architecture, including high polyunsaturated fatty acid content, distinct protein composition, and cholesterol/phospholipid ratios [4]. Cryosurvival is shaped by multiple, interacting factors—the cooling program, equilibration temperature and duration, extender chemistry, and the choice of cryoprotectants and additives [5,6]. Semen extenders play a pivotal protective role against cryopreservation-induced thermal and osmotic shock, oxidative stress, and ice-crystal injury by maintaining favorable pH, supplying energy substrates, providing antioxidant protection, and mitigating sperm cryoinjury [5]. Both natural (egg yolk, skim milk, soybean lecithin) and synthetic formulations are used, the latter allowing a tighter control of composition and performance [6,7].

Extenders are formulated to match species-specific semen characteristics and sensitivities. While glycerol is standard in mammalian semen, it is problematic in birds, prompting the use of alternative permeating cryoprotectants such as dimethylacetamide (DMA), dimethylformamide (DMF), or N-methylacetamide [8,9,10,11,12]. Because oxidative stress is a central driver of cryoinjury, avian extenders also frequently incorporate antioxidants with mixed efficacy across species and study designs [13,14,15]. There is also a large variation in other additives, such as energy substrates, antibiotics, and plant-based bioactive compounds, which may affect the final efficiency of poultry semen extenders. Despite a substantial literature on turkey extenders [16,17,18,19,20,21,22], individual protocol elements remain heterogeneous, and species- or breed-tuned optimization is incomplete.

Equilibration—a brief, low-temperature holding period before freezing—can critically affect the post-thaw function by permitting solute exchange while curbing metabolism [23,24]. Low-temperature holding also allows interactions between sperm, seminal plasma, and extender components (including cryoprotectants, antifreeze agents, antioxidants, and antibiotics) that may improve cryo-resilience [25]. In birds, optimal equilibration times are generally shorter than in mammals, yet they vary with extender and storage temperature; both too little and too much time can degrade outcomes through inadequate adaptation or energy depletion [26,27,28]. Antimicrobial agents likewise require contact time to act on contaminants, creating a potential trade-off between bacteriological control and sperm function.

Previous cryopreservation studies on turkey semen focus on one element at a time (e.g., extender or equilibration), report limited functional endpoints, or omit bacteriology which limits the ability to derive actionable settings for cryobanks. Here, we address this gap by (a) testing four extenders—Beltsville, Sperm Motility Medium (SMM), Botucrio, and Kobidil^+^—each paired with two equilibration times (20 vs. 40 min); (b) evaluating a multi-axis outcome panel that spans motility/kinematics, membrane and acrosome integrity, mitochondrial activity, apoptosis/necrosis, oxidative status, DNA fragmentation, and bacterial load; and (c) applying a standardized storage interval with immediate post-thaw assessment. This extender × time design provides a practical, head-to-head comparison that exposes interaction effects often missed in single-factor studies and, to our knowledge, is among the first in turkeys to integrate bacteriology alongside functional and oxidative/DNA endpoints within the same experiment.

We hypothesized that the extenders would differ in their optimal equilibration time and that this interaction would be reflected concordantly across functional, mitochondrial, oxidative, DNA, and bacteriological outputs. Our results identify time-specific best performers, offering clear, protocol-level guidance for turkey semen cryopreservation.

## 2. Materials and Methods

### 2.1. Sample Collection

Semen samples were obtained from the turkey breeding company Branko Nitra, a.s. (Nitra, Slovakia). The samples were collected through cloacal massage from 40 adult Big 6 males (35–55 weeks old) and transported to the laboratory in a thermal container (37 °C; M&G Int, Renate, Italy) within 30 min. All the ejaculates went through pre-evaluation for minimum quality criteria before the subsequent analysis. Animal handling procedures complied with the ethical guidelines of the Slovak Animal Protection Regulation RD 377/12, in accordance with European Directive 2010/63/EU.

Each ejaculate was divided into 5 equal aliquots, and each aliquot was diluted with extenders using the dilution ratio of 1:50–1:70 according to the initial sperm concentration. The 5 experimental treatments included the fresh sample as a negative control (diluted in Dulbecco’s Phosphate-Buffered Saline, Sigma-Aldrich, St. Louis, MO, USA; PBS), Beltsville (Agtech, Inc., Manhattan, KS, USA), SMM (Sperm Motility Medium; Slovak University of Agriculture, Nitra, Slovakia), Botucrio (Nidacon, Gotheburg, Sweden) and Kobidil^+^ (Minitube, Tiefenbach, Germany). Gentamicin was added to all experimental groups (0.25 g/L). The semen samples were subsequently dispersed into 0.5 mL straws which were cooled down at 4 °C for either 20 or 40 min, equilibrated over nitrogen vapors for 15 min, and stored in liquid nitrogen for one month. The semen samples from each treatment were pre-warmed at 37 °C prior to each assessment round for specific analysis and 100 µL of sample from each treatment was transferred into a sterile Eppendorf tube and kept at −20 °C for subsequent bacteriological examination [29,30].

### 2.2. Motility Evaluation

The computer-assisted sperm analysis (CASA) system (version 14.0 TOX IVOS II, Hamilton-Thorne Biosciences, Beverly, CA, USA) was used to evaluate the sperm motility as previously described [29]. Total and progressive motility were defined as the proportion of spermatozoa moving at a velocity greater than 5 μm/s and the proportion of spermatozoa moving at speeds higher than 20 μm/s, respectively. Secondary kinematic parameters, including path velocity (VAP; μm/s), linear velocity (VSL; μm/s), curvilinear velocity (VCL; μm/s), lateral amplitude of head displacement (ALH; μm) and beat cross frequency (BCF; Hz), straightness (STR; %), and linearity (LIN; %), were evaluated.

### 2.3. Sperm Membrane and Acrosome Integrity

A fluorescent staining protocol was used to assess the sperm membrane integrity. The cells were stained with CFDA (carboxylfluorescein diacetate; Sigma-Aldrich, St. Louis, MO, USA; 0.75 mg/mL in DMSO) for the quantification of cellular esterase activity as an indicator of cell viability. The staining protocol was PI (propidium iodide; Sigma-Aldrich, St. Louis, MO, USA; 5 μg/mL in PBS), as an indicator of dead cells, and the nucleic acid dye DAPI (4′6-diamidine-2-phenylindole; Sigma-Aldrich, St. Louis, MO, USA; 1 μmol/L in PBS) for counting the number of spermatozoa. The membrane integrity quantification was carried out by Glomax Multi+ (Promega, Madison, WI, USA). Acrosomal integrity was assessed by the PNA-lectin (FITC conjugate, Sigma-Aldrich, St. Louis, MO, USA; 10 μmol/L in PBS) staining procedure without a permeabilization step, leading to PNA binding to the surface of acrosome-reacted spermatozoa. Hence, PNA-positive cells were considered to be acrosome-reacted and were quantified by combined spectro-fluoro-luminometer Glomax Multi+ (Promega, Madison, WI, USA) [31,32].

### 2.4. Mitochondrial Membrane Potential

The mitochondrial membrane potential was evaluated by JC-1 assay kit (Cayman Chemical, Ann Arbor, MI, USA). Briefly, 100 μL of the sample was stained with 5 μL of JC-1 working solution and incubated for 30 min at 37 °C. Then, the samples were centrifuged for 5 min at 150× *g* at 25 °C and washed twice with a washing buffer provided by the commercial kit. Finally, the samples were transferred to a dark 96-chamber plate and analyzed by a combined GloMax-Multi+ Spectro-fluoro-luminometer (Promega, Madison, WI, USA) [33]. The resulting ∆Ψm was expressed as the ratio of JC-1 complexes to JC-1 monomers (green/red ratio).

### 2.5. Mitochondrial Activity

Mitochondrial metabolic activity was assessed through Mitochondrial Toxicity Test (MTT). For this, 20 μL of tetrazolium salt (Sigma-Aldrich, St. Louis, MO, USA) was dissolved in PBS (Dulbecco’s Phosphate-Buffered Saline without calcium chloride and magnesium chloride; Sigma-Aldrich, St. Louis, MO, USA) at a concentration of 5 mg/mL and added to each sample and incubated for 1 h at 37 °C. Subsequently, formazan crystals were dissolved using 80 μL of isopropanol (propan-2-ol; Centralchem, Bratislava, Slovakia). Optical density was measured by GloMax-Multi+ (Promega Corporation, Madison, WI, USA) at wavelength of 570 nm against 620 nm as reference. The results were expressed as a percentage of the control group set to 100% [34].

### 2.6. Superoxide Production

The nitroblue-tetrazolium (NBT) test was used for the quantification of intracellular superoxide radicals. The NBT salt was dissolved in PBS containing 1.5% DMSO (dimethyl sulfoxide, Sigma-Aldrich, St. Louis, MO, USA) to a final concentration of 1 mg/mL and added to the cells (100 µL per well). After 1 h of incubation (shaking at 37 °C with 95% air atmosphere and 5% CO_2_), the cells were washed twice with PBS and centrifuged at 300× *g* for 10 min. Then, the cells and formazan crystals were dissolved in 2 M KOH (potassium hydroxide; Centralchem, Bratislava, Slovakia) in DMSO. Optical density was determined at wavelength of 620 nm against 570 nm as reference by Glomax micro-plate reader Multi+ Software v2.3.2 (firmware v4.15) (Promega Corporation, Madison, WI, USA). Data was expressed in percentages of the native control set to 100% [35].

### 2.7. Sperm Chromatin Structure Assay

The sperm DNA damage was assessed with the sperm chromatin structure assay (SCSA) [36]. The samples were processed by the protocol described by Januskauskas and Johannisson [37]. Each sample was adjusted to 2 × 10^6^ sperm/mL with TNE buffer (0.15 M NaCl, 0.01 M Tris-HCl, 1 mM EDTA, pH 7.4; Sigma-Aldrich, St. Louis, MO, USA), mixed with 0.4 mL of acid detergent (0.17% Triton X-100, 0.15 M NaCl, and 0.08 N HCl, pH 1.4; Sigma-Aldrich, St. Louis, MO, USA). Following 30 s, the cells were stained with acridine orange solution (0.1 m citric acid, 0.2 M Na2HPO4, 1 mM EDTA, 0.15 M NaCl, pH 6.0; 6 μg/mL acridine orange; Sigma-Aldrich, St. Louis, MO, USA). The stained samples were transferred to a 96-black-well plate and analyzed using the GloMax-Multi+ combined spectro-fluoro-luminometer using appropriate filters to detect double-stranded DNA green fluorescence (530 ± 30 nm) and single-stranded DNA red fluorescence (>630 nm).

### 2.8. Apoptotic Spermatozoa

The externalization of phosphatidylserine as an index of sperm apoptosis was quantified through the Annexin V- FLUOS kit (Roche, Basel, Switzerland) in combination with propidium iodide (PI) to differentiate apoptotic and necrotic cells. The samples were washed in binding buffer and adjusted to a concentration of 1 × 10^6^ spermatozoa/mL. Annexin V (5 μL) was added to 100 μL of the suspension and incubated at room temperature for 20 min, followed by the addition of 5 μL PI and further incubation for at least 10 min. The stained samples were analyzed using the GloMax-Multi+ spectro-fluoro-luminometer (Promega Corporation, Madison, WI, USA). Spermatozoa were classified into three subpopulations: (1) viable (AV^−^/PI^−^), (2) apoptotic (AV^+^/PI^−^), and (3) necrotic (AV^−^/PI^+^), with results expressed as percentages [38].

### 2.9. ROS Measurement

The ROS concentration in the semen samples was measured using chemiluminescence procedure described by Lenický and Slanina [29]. The luminescent signal produced through the interaction of spermatozoa and luminol (Sigma-Aldrich, St. Louis, MO, USA) was quantified by GloMax-Multi+ spectro-fluoro-luminometer. The results were expressed as relative light units (RLU)/s/10^6^ spermatozoa.

### 2.10. Bacteriology

100 µL of each sample was inoculated into Tryptic soy agar (TSA, Soyabean Casein Digest Agar; Merck, Darmstadt, Germany) or blood agar (BA, Blood Agar Base No. 2; Merck, Darmstadt, Germany) under conditions described by Kačániová et al. [39] for the identification and quantification of the bacterial colonies present in semen specimens. Identification of bacterial species was performed by matrix-assisted laser desorption/ionization time-of-flight (MALDI-TOF) Biotyper mass spectrometry (Bruker Daltonics, Bremen, Germany) with procedure described by Lenický et al. [29] using Microflex LT instrument, flexControl software version 3.4, and MALDI Biotyper Bruker Taxonomy database (Bruker Daltonics, Bremen, Germany).

### 2.11. Statistical Analysis

Data were analyzed by the GLM procedure of SAS 9.4M9 (TS1M9) software in a 2 × 5 factorial arrangement. The model included the fixed effects of equilibration time (20 and 40 min), type of extender (fresh semen as negative control and 4 extenders) and their interaction effects. Least-square means were computed and tested for differences by Tukey’s test. Difference between least-squared means was considered to be significant at *p* < 0.05.

## 3. Results

### 3.1. Sperm Motility Characteristics

Sperm motility characteristics were affected by equilibration time regardless of ex-tender type (Table 1). Across the extenders, the post-thaw total motility was higher after 20 min of equilibration than after 40 min. Progressive motility and path velocity (VAP) also tended to be higher at 20 min, although these differences did not reach statistical significance. Likewise, 20 min equilibration yielded higher progressive velocity (VSL) and greater lateral head displacement (ALH) irrespective of extender.

Collapsing across equilibration times, Beltsville yielded the highest values for total motility, progressive motility, VAP (path velocity), VSL (progressive velocity), VCL (track speed), BCF (beat frequency), STR (straightness), and LIN (linearity), followed by SMM, Botucrio, and Kobidil^+^. In contrast, for ALH (lateral head displacement), Kobidil^+^ ranked highest, followed by Beltsville, SMM, and Botucrio (Table 2).

All motility characteristics (except for VCL and BCF) exhibited a significant extender × equilibration-time interaction (Table 3). At 20 min, Beltsville delivered the highest motility metrics; at 40 min, SMM performed best, though some differences were not statistically significant. For ALH, Kobidil^+^ ranked highest at both 20 and 40 min, but the advantage at 40 min did not reach significance.

### 3.2. Sperm Quality Characteristics

As shown in Table 4, 20 min of equilibration improved membrane and acrosome integrity and increased mitochondrial membrane potential, while reducing non-viable sperm, DNA fragmentation, and ROS levels when compared with 40 min.

Collapsed across equilibration times, Beltsville yielded the highest membrane and acrosome integrity, mitochondrial membrane potential (ΔΨm), and mitochondrial activity, followed by SMM, Botucrio, and Kobidil^+^ (Table 5). Conversely, apoptotic and non-viable sperm proportions, DNA fragmentation, and both ROS and superoxide levels were lowest with Beltsville, then SMM, Botucrio, and Kobidil^+^.

All sperm quality outcomes showed a significant extender × equilibration-time interaction (Table 6). For Beltsville, Botucrio, and Kobidil^+^, extending equilibration from 20 to 40 min reduced membrane and acrosome integrity, mitochondrial membrane potential (ΔΨm), and mitochondrial activity. In contrast, SMM improved with 40 min. The same pattern held for damage/stress markers: apoptosis, non-viability, DNA fragmentation, superoxide (NBT), and ROS were lower at 20 min with Beltsville/Botucrio/Kobidil^+^, whereas SMM yielded lower values at 40 min.

### 3.3. Bacterial Load

**Figure 1:**
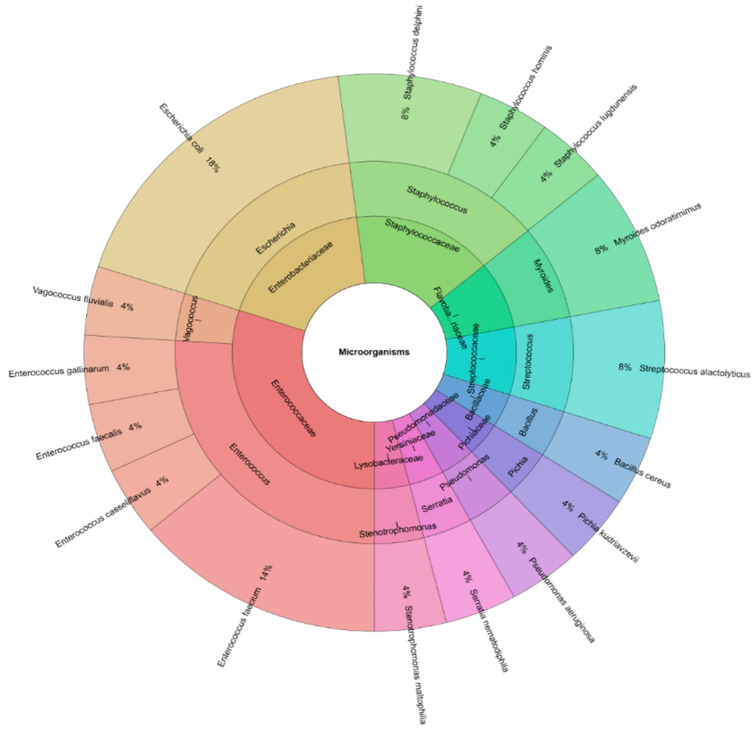
Krona chart of the identified bacterial species by MALDI-TOF MS Biotyper recovered from turkey fresh semen samples (outermost ring: species, middle ring: genus, and innermost ring: family).

From the semen, 16 bacterial species were isolated, including *Escherichia coli*, *Staphylococcus delphini*, *Staphylococcus hominis*, *Staphylococcus lugdunensis*, *Myroides odoratimimus*, *Streptococcus alactolyticus*, *Bacillus cereus*, *Pichia kudriavzevii*, *Pseudomonas aeruginosa*, *Serratia nematodiphila*, *Stenotrophomonas maltophilia*, *Enterococcus faecium*, *Enterococcus casseliflavus*, *Enterococcus faecalis*, *Enterococcus gallinarum*, and *Vagococcus fluvialis* (Figure 1). These species belonged to 11 genera and 10 families.

Across extenders, 40 min of equilibration yielded lower bacterial count on tryptic soy agar than 20 min (Table 4). All extenders reduced bacterial load versus fresh semen, but Kobidil^+^ showed the weakest inhibition (Table 5). Total bacterial counts showed no significant extender × time interaction. By species, Botucrio inhibited all identified taxa except *B. cereus* and *S. maltophilia*, which remained resistant both times (Table 7). Kobidil^+^ showed activity only against *B. cereus* at 20 and 40 min. SMM inhibited *E. coli*, *M. odoratimimus*, and *S. nematodiphila*; other species were unaffected both times. Beltsville displayed broad activity: of 16 species detected, only six (*E. coli*, *M. odoratimimus*, *P. aeruginosa*, *S. nematodiphila*, *S. hominis*, and *S. maltophilia*) remained resistant at both equilibration intervals.

## 4. Discussion

This study shows that post-thaw turkey semen quality is governed by a clear extender × equilibration-time interaction. Collapsing across endpoints, 20 min at 4 °C outperformed 40 min for Beltsville, Botucrio, and Kobidil^+^, improving motility/kinematics, membrane and acrosome integrity, mitochondrial membrane potential and activity, and reducing non-viable cells, DNA fragmentation, and ROS; in contrast, SMM achieved its best performance at 40 min. Although 40 min reduced total bacteria load, this bacteriological advantage was accompanied by inferior sperm function relative to the optimal time for each extender. Together, these data argue that turkey semen cryopreservation requires time-specific optimization for each extender, rather than a single holding time for all formulations.

The strategic value of semen cryopreservation for genetic resource management is well established [40]. Extenders are central to cryosurvival because they buffer pH, supply substrates, mitigate osmotic/thermal stress and oxidative damage, and limit microbial growth [5,6]. These properties derive from diverse metabolites and components, both synthetic and natural, which vary according to the extender type, preservation method, and species of interest. Avian semen is particularly sensitive to cryopreservation as opposed to mammalian ejaculates, largely due to intracellular ice crystallization during freezing that can markedly impair sperm quality. In the case of cryo-induced oxidative stress, antioxidant supplementation in avian extenders - whether natural or synthetic, has produced mixed outcomes for cooled and frozen rooster semen, with reported effects on motility, membrane integrity, viability, and fertility [41]. The sperm cell generates ATP via oxidative phosphorylation and glycolysis, and both pathways support normal function; however, substrate use is species-dependent. Although mitochondrial respiration is more ATP-efficient overall, glycolysis predominates in the distal flagellum, where limited ATP diffusion from mitochondria makes local glycolytic ATP especially important for motility [42]. Consistent with this, carbohydrate choice matters: different sugars used as energy substrates yield distinct post-thaw quality profiles [43,44], and several sugars provide dual benefits by serving as osmoprotective/cryoprotective agents that mitigate freezing-induced damage in addition to fueling metabolism [44,45]. Within this context, Beltsville has a track record in poultry [46,47,48] and a defined ionic/energy profile [49]. Botucrio and Kobidil^+^ were originally designed for stallion and boar semen, respectively [50,51,52,53,54], while the SMM is a newly developed extender at the Slovak University of Agriculture in Nitra (patent no. 289201) containing saline with an osmolarity of 270–320 mOsm·L^−1^ and pH 5.5–7.0, and is supplemented with D-levulose, α-D-glucopyranosyl-α-D-glucopyranoside, 1,3,7-trimethylxanthine, and 2-aminoethanesulfonic acid. The differences in type, number and proportion of cryoprotectants, energy substrates, antibiotics, antioxidants, and other constituents of extenders used in this work might be primarily responsible for their different cryo-efficiency. Out of the chosen extenders, Beltsville seems to present with the most optimal balance of ingredients for the preservation of turkey sperm quality during the freeze–thaw process.

During equilibration, holding spermatozoa at 4–5 °C lowers metabolic demand and permits controlled osmotic re-equilibration as cells first shrink in a hyperosmotic medium and then re-expand as permeating solutes enter [55]. In birds, whose filiform sperm and PUFA-rich membranes increase cold and osmotic sensitivity, the optimal window is typically minutes [8,56], not hours as is the case of mammals [57]. Prior studies illustrate that the “right” duration depends on the cryoprotectant/extender system: with DMSO or DMA, effective windows can be as short as 10 min and even ≈2 min in some poultry protocols [28,58,59,60], while small changes (e.g., 5 min vs. 1 min) can already shift outcomes with turkey extenders such as Tselutin [61]. Conversely, varying equilibration from 0 to 2 h in a Ringer’s-lactate/egg-yolk/DMSO system did not change motility/viability, though membrane integrity benefited at 2 h [28], underscoring that different formulations engage different limiting steps [62,63]. Indeed, CPA identity and molecular size govern membrane crossing rates and thus the time needed to achieve protective intracellular/extracellular balance—larger or less permeant solutes typically require longer [64], while short, DMA-, or DMSO-based systems may require shorter [58,59,60]. Moreover, time at low temperature is not neutral: prolonged holds can worsen phase transitions, ATP drain, and redox stress in avian sperm, so any bacteriological or osmotic gains must be weighed against physiology costs. Our contribution is to show, in one unified experiment, that these kinetics are extender-specific for turkey semen: Beltsville, Botucrio, and Kobidil^+^ were optimal at 20 min, whereas SMM required 40 min to express its full protective effect. The 20 min advantage for Beltsville (a poultry-oriented matrix) fits the avian precedent of brief equilibration [8,56] and suggests that for these three formulations the dominant risks are time-dependent injury (cold/oxidative, energy depletion), which is minimized by a shorter hold. In contrast, SMM’s 40 min optimum indicates that its protective components (cryoprotectants and/or macromolecular additives) are likely to exhibit slower membrane interaction or binding kinetics, needing additional contact time to reach a favorable intracellular/extracellular distribution, after which mitochondrial potential improves and ROS/DNA damage declines. Importantly, this supports earlier reports that very short equilibration (e.g., 5 min) does not itself explain cryo-losses in turkeys using other CPA systems [63]; rather, the CPA–extender pair sets the time requirement, and deviating from that optimum either leaves cells under-equilibrated (osmotic stress) or over-exposed to chilling-associated injury. Taken together, we may agree with the concept that equilibration is a kinetics matching problem: equilibration time has to be carefully chosen so that it matches the permeability and binding properties of the specific extender/CPA (Beltsville/Botucrio/Kobidil^+^ ≈ 20 min; SMM ≈ 40 min) while minimizing the negative effects of low temperatures, which is fully consistent with the species- and formulation-dependent patterns documented in poultry [28,58,59,60,61,64].

Longer equilibration (40 min) uniformly decreased the bacterial load, indicating more time for antimicrobial action, yet it also degraded sperm quality relative to the optimal (shorter) times for Beltsville/Botucrio/Kobidil^+^. Extenders differed in spectrum: Botucrio broadly inhibited most species except *B. cereus* and *S. maltophilia*; Kobidil^+^ showed activity mainly against *B. cereus*; SMM inhibited *E. coli*, *M. odoratimimus*, and *S. nematodiphila*; Beltsville suppressed most species but not *E. coli*. These patterns are consistent with antibiotic class/dose differences, initial semen microbiota, and species-specific sperm tolerance to antibiotics [65]. Practically, our data favor choosing the extender for sperm protection and then targeting residual organisms (e.g., supplementing Beltsville with anti-*E. coli* coverage) rather than extending the holding time to 40 min as a blanket antimicrobial strategy.

Overall, we may conclude that for routine cryobanking Beltsville at 20 min is the default setting—delivering robust improvements in motility, membrane integrity, mitochondrial membrane potential, and DNA integrity. If SMM is used, a 40 min equilibration should be adopted to realize its protective effect. For microbiological control, the extender’s antimicrobial spectrum should be prioritized and target specific antibiotics rather than lengthening the holding time, which compromises the sperm function. Together, these protocol-level choices are immediately actionable for AI programs seeking reliable, reproducible post-thaw performance.

Finally, the weaknesses of this study should be acknowledged. Our experimental approach is limited by the absence of fertility endpoints (e.g., hatchability) and the inability to isolate antibiotic effects from the base extender matrix; prior reports also show that equilibration efficacy can depend on CPA identity and molecular size [63,64]. Future work should therefore (a) map time windows more finely (e.g., 10–30 min for Beltsville; 30–50 min for SMM), (b) vary antibiotic spectra/doses within a single base medium, and (c) link laboratory findings to field fertility.

## 5. Conclusions

Turkey sperm cryosurvival is not governed by a single “best” equilibration time, but by a time–formulation match. Beltsville at 20 min and SMM at 40 min emerge as time-specific best performers, while lengthening the hold to reduce bacteria imposes a threat to the post-thaw sperm viability. Centering protocols on this interaction and adding targeted antimicrobial coverage where needed offers a practical path to more reliable turkey semen biobanking.

## Figures and Tables

**Table 1:** Effect of equilibration time on sperm post-thaw motility characteristics regardless of extender type.

Storage Time (min)	20	40	SEM ^1^	*p*-Value
MOT (%)	31.46 ^a^	27.30 ^b^	1.08	0.008
PROG (%)	19.70	18.10	0.65	0.08
VAP (μm/s)	37.50	35.54	1.06	0.19
VSL (μm/s)	26.34 ^a^	21.80 ^b^	0.95	0.001
VCL (μm/s)	66.88	64.34	1.54	0.25
ALH (μm)	4.05 ^a^	3.58 ^b^	0.075	0.0001
BCF (Hz)	27.58	27.30	0.87	0.82
STR (%)	45.36	45.66	0.75	0.77
LIN (%)	20.40	19.16	0.75	0.25

^1^ Standard Error of the Mean; values in columns with different letters differ significantly. MOT—motility; PROG—progressive motility; VAP—path velocity; VSL—progressive velocity; VCL—track speed; ALH—lateral amplitude; BCF—beat frequency; STR—straightness; LIN—linearity.

**Table 2:** Effect of selected extenders on sperm post-thaw motility characteristics regardless of equilibration time.

Extender	Fresh Semen	Beltsville	SMM ^1^	Botucrio	Kobidil^+^	SEM ^2^	*p*-Value
MOT (%)	77.66 ^a^	31.66 ^b^	18.58 ^c^	12.91 ^d^	6.08 ^c^	1.70	0.0001
PROG (%)	58.16 ^a^	18.25 ^b^	12.58 ^c^	1.91 ^d^	3.58 ^d^	1.03	0.0001
VAP (μm/s)	52.86 ^a^	41.82 ^b^	37.86 ^bc^	17.57 ^d^	32.49 ^c^	1.68	0.0001
VSL (μm/s)	36.90 ^a^	29.11 ^b^	27.20 ^b^	11.85 ^c^	15.30 ^c^	1.51	0.0001
VCL (μm/s)	95.55 ^a^	72.45 ^b^	65.48 ^bc^	35.93 ^d^	58.64 ^cd^	2.44	0.0001
ALH (μm)	5.20 ^a^	4.05 ^c^	3.93 ^c^	1.32 ^d^	4.59 ^b^	0.11	0.0001
BCF (Hz)	34.45 ^a^	28.18 ^b^	26.54 ^b^	19.05 ^c^	28.97 ^b^	1.38	0.0001
STR (%)	62.83 ^a^	57.91 ^b^	45.58 ^c^	22.4 ^e^	38.83 ^d^	1.19	0.0001
LIN (%)	36.33 ^a^	24.25 ^b^	19.83 ^b^	6.83 ^d^	11.66 ^c^	1.19	0.0001

^1^ SMMW: Sperm Motility Medium. ^2^ Standard Error of the Mean; values in columns with different letters differ significantly. MOT—motility; PROG—progressive motility; VAP—path velocity; VSL—progressive velocity; VCL—track speed; ALH—lateral amplitude; BCF—beat frequency; STR—straightness; LIN—linearity.

**Table 3:** Interaction of equilibration time and type of extender on sperm post-thaw motility characteristics.

Storage Time (min)	20					40					SEM ^2^	*p*-Value
Type of Extender	Fresh	Beltsville	SMM ^1^	Botucrio	Kobidil^+^	Fresh	Beltsville	SMM ^1^	Botucrio	Kobidil^+^		
MOT (%)	77.66 ^a^	45.00 ^b^	12.00 ^d^	7.00 ^d^	15.66 ^cd^	77.66 ^a^	18.33 ^cd^	25.16 ^c^	5.16 ^d^	10.16 ^d^	2.41	0.0001
PROG (%)	58.16 ^a^	22.66 ^b^	11.00 ^cd^	2.16 ^e^	4.50 ^de^	58.16 ^a^	13.83 ^c^	14.16 ^c^	1.66 ^e^	2.66 ^e^	1.45	0.002
VAP (μm/s)	52.86 ^a^	44.46 ^a^	34.35 ^bc^	22 ^de^	33.83 ^bc^	52.86 ^a^	39.18 ^bc^	41.38 ^ab^	13.15 ^e^	31.15 ^cd^	2.37	0.02
VSL (μm/s)	36.90 ^a^	31.73 ^ab^	26.21 ^bcd^	17.81 ^de^	19.06 ^cde^	36.90 ^a^	26.50 ^bcd^	28.18 ^abc^	5.88 ^f^	11.55 ^ef^	2.14	0.01
VCL (μm/s)	95.55	76.65	62.26	37.50	62.43	95.55	68.25	68.70	34.36	54.85	3.46	0.20
ALH (μm)	5.20 ^a^	4.11 ^c^	3.86 ^c^	2.15 ^d^	4.93 ^ab^	5.20 ^a^	3.98 ^c^	4.00 ^c^	0.50 ^e^	4.25 ^bc^	0.16	0.0001
BCF (Hz)	34.45	28.48	25.50	20.01	29.45	34.45	27.88	27.58	18.10	28.50	1.96	0.88
STR (%)	62.83 ^a^	60.66 ^ab^	37.83 ^c^	26.16 ^d^	39.33 ^c^	62.83 ^a^	55.16 ^ab^	53.33 ^b^	18.66 ^d^	38.33 ^c^	1.68	0.0001
LIN (%)	36.33 ^a^	27.00 ^b^	17.33 ^cd^	7.66 ^e^	13.66 ^de^	36.33 ^a^	21.50 ^bc^	23.33 ^bc^	6.00 ^e^	9.66 ^de^	1.69	0.03

^1^ SMM: Sperm Motility Medium. ^2^ Standard Error of the Mean; values in columns with different letters differ significantly. MOT—motility; PROG—progressive motility; VAP—path velocity; VSL—progressive velocity; VCL—track speed; ALH—lateral amplitude; BCF—beat frequency; STR—straightness; LIN—linearity.

**Table 4:** Effects of equilibration time on sperm post-thaw quality characteristics.

Storage Time (Min)	20	40	SEM ^1^	*p*-Value
Membrane integrity (%)	45.27 ^a^	41.70 ^b^	0.054	0.0001
Acrosome integrity (%)	53.33 ^a^	50.98 ^b^	0.55	0.0044
Apoptosis (%)	38.34	39.14	0.40	0.16
Non-viable sperm (Propidium Iodide test; %)	23.76 ^b^	26.62 ^a^	0.45	0.0001
DNA Fragmentation (SCSA) (%)	34.21 ^b^	35.83 ^a^	0.44	0.01
MMP (green/red ratio)	0.38 ^a^	0.36 ^b^	0.005	0.009
Mitochondrial activity (%)	50.89	49.55	1.02	0.35
Superoxide production (%)	143.33	140.94	1.06	0.11
ROS (RLU/s/10^6^ sperm)	16.29 ^b^	16.95 ^a^	0.18	0.01
TSA-Bacterial count (log10 CFU/mL)	2.14 ^a^	1.82 ^b^	0.071	0.0023
BA-Bacterial count (log10 CFU/mL)	1.82	1.54	0.12	0.11

^1^ Standard Error of the Mean; values in columns with different letters differ significantly. MMP—mitochondrial membrane potential; ROS—reactive oxygen species; TSA—tryptic soy agar; BA—blood agar.

**Table 5:** Effects of type of extender on sperm post-thaw quality characteristics.

Type of Extender	Fresh	Beltsville	SMM ^1^	Botucrio	Kobidil^+^	SEM ^2^	*p*-Value
Membrane integrity (%)	82.09 ^a^	53.24 ^b^	36.68 ^c^	24.61 ^d^	20.80 ^e^	0.85	0.0001
Acrosome integrity (%)	88.11 ^a^	59.81 ^b^	50.67 ^c^	38.66 ^d^	23.52 ^e^	0.87	0.0001
Apoptosis (%)	18.58 ^e^	36.14 ^d^	39.61 ^c^	46.11 ^b^	53.37 ^a^	0.63	0.0001
Non-viable sperm (Propidium Iodide test; %)	3.40 ^e^	22.02 ^d^	25.39 ^c^	34.80 ^b^	40.35 ^a^	0.71	0.0001
DNA Fragmentation (%)	11.80 ^e^	34.94 ^d^	38.10 ^c^	47.80 ^a^	42.45 ^b^	0.69	0.0001
MMP (green/red ratio)	0.72^a^	0.43 ^b^	0.34 ^c^	0.14 ^e^	0.20 ^d^	0.008	0.0001
Mitochondrial activity (%)	99.99 ^a^	56.05 ^b^	50.29 ^b^	14.65 ^d^	30.4 ^c^	1.61	0.0001
Superoxide production (%)	100.00 ^e^	129.16 ^d^	140.91 ^c^	179.65 ^a^	160.97 ^b^	1.67	0.0001
ROS (RLU/s/10^6^ sperm)	4.61 ^e^	13.87 ^d^	16.16 ^c^	26.84 ^a^	21.63 ^b^	0.29	0.0001
TSA-Bacterial count (log10 CFU/mL)	3.34 ^a^	1.60 ^c^	1.46 ^c^	1.23 ^c^	2.28 ^b^	0.11	0.0001
BA-Bacterial count (log10 CFU/mL)	3.17 ^a^	1.28 ^bc^	1.19 ^bc^	0.90 ^c^	1.84 ^b^	0.19	0.0001

^1^ SMM: Sperm Motility Medium. ^2^ Standard Error of the Mean; values in columns with different letters differ significantly. MMP—mitochondrial membrane potential; ROS—reactive oxygen species; TSA—tryptic soy agar; BA—blood agar.

**Table 6:** Interaction of equilibration time and type of extender on sperm post-thaw quality characteristics.

Storage Time (min)	20					40					SEM ^2^	*p*-Value
Type of Extender	Fresh	Beltsville	SMM ^1^	Botucrio	Kobidil^+^	Fresh	Beltsville	SMM ^1^	Botucrio	Kobidil^+^		
Membrane integrity (%)	82.09 ^a^	62.81 ^b^	32.71 ^d^	25.47 ^e^	23.29 ^ef^	82.09 ^a^	43.67 ^c^	40.65 ^c^	23.76 ^ef^	18.31 ^f^	1.21	0.0001
Acrosome integrity (%)	88.11 ^a^	65.61 ^b^	45.58 ^d^	42.76 ^d^	24.58 ^f^	88.11 ^a^	54.01 ^c^	55.76 ^c^	34.56 ^e^	22.46 ^f^	1.24	0.0001
Apoptosis (%)	18.58 ^e^	34.61 ^d^	42.35 ^c^	44.76 ^bc^	51.41 ^a^	18.58 ^e^	37.66 ^d^	36.88 ^d^	47.25 ^b^	55.33 ^a^	0.89	0.0001
Non-viable sperm (%)	3.40 ^f^	18.18 ^e^	27.73 ^c^	34.11 ^b^	35.40 ^b^	3.40 ^f^	25.86 ^cd^	23.05 ^d^	25.48 ^b^	45.30 ^a^	1.01	0.0001
DNA Fragmentation (%)	11.80 ^f^	31.15 ^e^	40.06 ^cd^	46.11 ^ab^	41.91 ^bc^	11.80 ^f^	38.73 ^cd^	36.15 ^d^	49.48 ^a^	42.98 ^bc^	0.98	0.0001
MMP (green/red ratio)	0.72 ^a^	0.53 ^b^	0.25 ^e^	0.15 ^g^	0.23 ^ef^	0.72 ^a^	0.34 ^d^	0.42 ^c^	0.13 ^g^	0.18 ^fg^	0.01	0.0001
Mitochondrial activity (%)	99.99 ^a^	58.47 ^b^	44.72 ^cd^	16.28 ^fg^	35.00 ^de^	99.99 ^a^	53.64 ^bc^	55.86 ^b^	13.02 ^g^	13.02 ^g^	2.28	0.0006
Superoxide production (%)	100.00 ^g^	121.62 ^f^	150.78 ^d^	175.00 ^ab^	157.32 ^cd^	100.00 ^g^	136.70 ^e^	131.04 ^ef^	184.31 ^a^	164.62 ^bc^	2.37	0.0001
ROS (RLU/s/10^6^ sperm)	4.61 ^f^	12.60 ^e^	19.19 ^c^	25.75 ^b^	19.32 ^c^	4.61 ^f^	15.15 ^d^	13.13 ^e^	27.92 ^a^	23.95 ^b^	0.41	0.0001
TSA-Bacterial count (log10 CFU/mL)	3.34	1.82	1.58	1.35	2.63	3.34	1.39	1.34	1.11	1.93	1.11	0.26
BA-Bacterial count (log10 CFU/mL)	3.17	1.37	1.26	1.11	2.21	3.17	1.20	1.12	0.72	1.47	0.27	0.71

^1^ SMM: Sperm Motility Medium. ^2^ Standard Error of the Mean; values in columns with different letters differ significantly. MMP—mitochondrial membrane potential; ROS—reactive oxygen species; TSA—tryptic soy agar; BA—blood agar.

**Table 7:** Bacterial strain resistance and sensitivity affected by interaction of equilibration time and type of extender.

Storage Time (Min)	20									40								
Type of Extender	Fresh Semen	Beltsville	%	SMM ^1^	%	Botucrio	%	Kobidil^+^	%	Fresh Semen	Beltsville	%	SMM ^1^	%	Botucrio	%	Kobidil^+^	%
*Bacillus cereus*	R	S	0	R	6	R	50	S	0	R	S	0	R	7.1	R	50	S	0
*Enterococcus casseliflavus*	R	S	0	R	6	S	0	R	4	R	S	0	R	7.1	S	0	R	5
*Enterococcus faecalis*	R	S	0	R	6	S	0	R	4	R	S	0	R	7.1	S	0	R	5
*Enterococcus faecium*	R	S	0	R	13	S	0	R	13	R	S	0	R	14.3	S	0	R	11
*Enterococcus gallinarum*	R	S	0	R	6	S	0	R	4	R	S	0	R	7.1	S	0	R	5
*Escherichia coli*	R	R	38	S	0	S	0	R	17	R	R	17	S	0	S	0	R	16
*Myroides odoratimimus*	R	R	13	S	0	S	0	R	9	R	R	17	S	0	S	0	R	5
*Pichia kudriavzevii*	R	S	0	R	6	S	0	R	4	R	S	0	R	7.1	S	0	R	5
*Pseudomonas aeruginosa*	R	R	13	R	6	S	0	R	4	R	R	17	R	7.1	S	0	R	5
*Serratia nematodiphila*	R	R	13	S	0	S	0	R	4	R	R	17	S	0	S	0	R	5
*Staphylococcus delphini*	R	S	0	R	13	S	0	R	9	R	S	0	R	7.1	S	0	R	11
*Staphylococcus hominis*	R	R	13	R	6	S	0	R	4	R	R	17	R	7.1	S	0	R	5
*Staphylococcus lugdunensis*	R	S	0	R	6	S	0	R	4	R	S	0	R	7.1	S	0	R	5
*Stenotrophomonas maltophilia*	R	R	13	R	6	R	50	R	4	R	R	17	R	7.1	R	50	R	5
*Streptococcus alactolyticus*	R	S	0	R	13	S	0	R	9	R	S	0	R	7.1	S	0	R	5
*Vagococcus fluvialis*	R	S	0	R	6	S	0	R	4	R	S	0	R	7.1	S	0	R	5
Bacterial Resistance (%)	100	37.5	100	81.25	100	12.5	100	6.25	100	100	37.5	100	81.25	100	12.5	100	6.25	100
Bacterial Sensitivity (%)	100	62.5	100	18.75	100	87.5	100	93.75	100	100	62.5	100	18.75	100	87.5	100	93.75	100

^1^ SMM: Sperm Motility Medium.

## Data Availability

The data are available and contained within the article, with further questions able to be referred to the corresponding author.

## References

[1] Asaduzzaman M., Miah A.G., Salma U., Jahan M.S. (2022). Efficiency of natural mating and artificial insemination in turkey (*Meleagris gallopavo*) breeding. Asian-Australas. J. Biosci. Biotechnol..

[2] Zong Y., Li Y., Sun Y., Mehaisen G.M., Ma T., Chen J. (2023). Chicken sperm cryopreservation: Review of techniques, freezing damage, and freezability mechanisms. Agriculture.

[3] Iaffaldano N., Di Iorio M., Cerolini S., Manchisi A. (2016). Overview of turkey semen storage: Focus on cryopreservation—A review. Ann. Anim. Sci..

[4] Partyka A., Niżański W. (2022). Advances in storage of poultry semen. Anim. Reprod. Sci..

[5] Bustani G.S., Baiee F.H. (2021). Semen extenders: An evaluative overview of preservative mechanisms of semen and semen extenders. Vet. World.

[6] Alkali I.M., Asuku S.O., Colombo M., Bukar M.M., Waziri M.A., Luvoni G.C. (2022). Spermatozoa survival in egg yolk-based and soybean-based extenders at ambient and chilling temperature in domestic turkeys (*Meleagris gallopavo*). Animals.

[7] Raziq F., Khan M., Ullah A., Farooq U., Akhtar R., Siddique B., Abbas G., Ali F., Aslam S., Khaliq H. (2025). Effects of various semen extenders on the hatching characteristics of Japanese quail. Braz. J. Poult. Sci..

[8] Blesbois E., Brillard J.-P. (2007). Specific features of in vivo and in vitro sperm storage in birds. Animal.

[9] Blanco J.M., Long J.A., Gee G., Wildt D.E., Donoghue A.M. (2012). Comparative cryopreservation of avian spermatozoa: Effects of freezing and thawing rates on turkey and sandhill crane sperm cryosurvival. Anim. Reprod. Sci..

[10] Thananurak P., Chuaychu-Noo N., Thélie A., Phasuk Y., Vongpralub T., Blesbois E. (2019). Sucrose increases the quality and fertilizing ability of cryopreserved chicken sperms in contrast to raffinose. Poult. Sci..

[11] Mosca F., Madeddu M., Sayed A.A., Zaniboni L., Iaffaldano N., Cerolini S. (2016). Combined effect of permeant and non-permeant cryoprotectants on the quality of frozen/thawed chicken sperm. Cryobiology.

[12] Mosca F., Zaniboni L., Sayed A.A., Madeddu M., Iaffaldano N., Cerolini S. (2019). Effect of dimethylacetamide and N-methylacetamide on the quality and fertility of frozen/thawed chicken semen. Poult. Sci..

[13] Aitken R.J., Wingate J.K., De Iuliis G.N., Koppers A.J., McLaughlin E.A. (2006). Cis-unsaturated fatty acids stimulate reactive oxygen species generation and lipid peroxidation in human spermatozoa. J. Clin. Endocrinol. Metab..

[14] Partyka A., Niżański W. (2021). Supplementation of avian semen extenders with antioxidants to improve semen quality—Is it an effective strategy?. Antioxidants.

[15] Ratchamak R., Authaida S., Koedkanmark T., Saiyamanon H., Boonkum W., Chankitisakul V. (2025). Optimization of rooster semen preservation: A comparative study of extender types and antioxidant supplementation strategies during cold storage. Poult. Sci..

[16] Di Iorio M., Rusco G., Iampietro R., Colonna M.A., Zaniboni L., Cerolini S., Iaffaldano N. (2020). Finding an effective freezing protocol for turkey semen: Benefits of ficoll as non-permeant cryoprotectant and 1:4 as dilution rate. Animals.

[17] Gloria A., Toscani T., Robbe D., Parrillo S., De Amicis I., Contri A. (2019). Cryopreservation of turkey spermatozoa without permeant cryoprotectants. Anim. Reprod. Sci..

[18] Blanco J.M., Long J.A., Gee G., Wildt D.E., Donoghue A.M. (2011). Comparative cryopreservation of avian spermatozoa: Benefits of non-permeating osmoprotectants and ATP on turkey and crane sperm cryosurvival. Anim. Reprod. Sci..

[19] Kuzlu M., Taskin A. (2017). The effect of different extenders on the sperm motility and viability of frozen turkey semen. Indian J. Anim. Res..

[20] Iaffaldano N., Rosato M., Manchisi A., Centoducati G., Meluzzi A. (2005). Comparison of different extenders on the quality characteristics of turkey semen during storage. Ital. J. Anim. Sci..

[21] Long J., Kramer M. (2003). Effect of vitamin E on lipid peroxidation and fertility after artificial insemination with liquid-stored turkey semen. Poult. Sci..

[22] Long J., Conn T. (2012). Use of phosphatidylcholine to improve the function of turkey semen stored at 4 °C for 24 hours. Poult. Sci..

[23] Thema M.A., Mkhize N.R., Sebopela M.D., Ledwaba M.R., Mphaphathi M.L. (2025). Effect of pre-freezing 18 °C holding time on post-thaw motility and morphometry of cryopreserved boar epididymal sperm. Animals.

[24] Yeste M., Estrada E., Rivera del Alamo M.-M., Bonet S., Rigau T., Rodriguez-Gil J.-E. (2014). The increase in phosphorylation levels of serine residues of protein HSP70 during holding time at 17 °C is concomitant with a higher cryotolerance of boar spermatozoa. PLoS ONE.

[25] Torres M.A., Monteiro M.S., Passarelli M.S., Papa F.O., Dell’Aqua Jr J.A., Alvarenga M.A., Martins S.M., de Andrade A.F. (2019). The ideal holding time for boar semen is 24 h at 17 °C prior to short-cryopreservation protocols. Cryobiology.

[26] Cassinelli C., Zaniboni L., Mangiagalli M., Cerolini S. (2008). Sperm quality changes during equilibration time at 4 °C before cryopreservation of chicken semen. Poult. Sci..

[27] Zaniboni L., Cassinelli C., Mangiagalli M.G., Gliozzi T.M., Cerolini S. (2014). Pellet cryopreservation for chicken semen: Effects of sperm working concentration, cryoprotectant concentration, and equilibration time during in vitro processing. Theriogenology.

[28] Wahjuningsih S., Arif A., Khaerudin P.H., Putri A. (2024). The effects of equilibration time and post-thawing temperatures in cryopreservation of gaga chicken semen. Adv. Anim. Vet. Sci..

[29] Lenický M., Slanina T., Kačániová M., Galovičová L., Petrovičová M., Ďuračka M., Benko F., Kováč J., Tvrdá E. (2021). Identification of bacterial profiles and their interactions with selected quality, oxidative, and immunological parameters of turkey semen. Animals.

[30] Tvrdá E., Petrovičová M., Benko F., Ďuračka M., Galovičová L., Slanina T., Kačániová M. (2022). Curcumin attenuates damage to rooster spermatozoa exposed to selected uropathogens. Pharmaceutics.

[31] Baňas Š., Benko F., Ďuračka M., Lukáč N., Tvrdá E. (2023). Epicatechin prevents cryocapacitation of bovine spermatozoa through antioxidant activity and stabilization of transmembrane ion channels. Int. J. Mol. Sci..

[32] Benko F., Mohammadi-Sangcheshmeh A., Ďuračka M., Lukáč N., Tvrdá E. (2022). In vitro versus cryo-induced capacitation of bovine spermatozoa, part 1: Structural, functional, and oxidative similarities and differences. PLoS ONE.

[33] Woelders H., De Wit A., Engel B., Hulsegge B., Grasseau I., Blesbois E., Bernal B., Santiago-Moreno J. (2022). Freezing chicken semen: Influence of base medium osmolality, cryoprotectants, cryoprotectant concentration, and cooling rate on post-thaw sperm survival. Cryobiology.

[34] Tvrdá E., Kováčik A., Tušimová E., Paál D., Mackovich A., Alimov J., Lukáč N. (2016). Antioxidant efficiency of lycopene on oxidative stress-induced damage in bovine spermatozoa. J. Anim. Sci. Biotechnol..

[35] Tvrdá E., Tušimová E., Kováčik A., Paál D., Libová Ľ., Lukáč N. (2016). Protective effects of quercetin on selected oxidative biomarkers in bovine spermatozoa subjected to ferrous ascorbate. Reprod. Domest. Anim..

[36] Evenson D.P. (2022). Sperm chromatin structure assay (SCSA^®^) for fertility assessment. Curr. Protoc..

[37] Januskauskas A., Johannisson A., Rodriguez-Martinez H. (2001). Assessment of sperm quality through fluorometry and sperm chromatin structure assay in relation to field fertility of frozen-thawed semen from Swedish AI bulls. Theriogenology.

[38] Najafi A., Mohammadi H., Sharifi S.D., Rahimi A. (2024). Apigenin supplementation substantially improves rooster sperm freezability and post-thaw function. Sci. Rep..

[39] Kačániová M., Terentjeva M., Štefániková J., Žiarovská J., Savitskaya T., Grinshpan D., Kowalczewski P.Ł., Vukovic N., Tvrdá E. (2020). Chemical composition and antimicrobial activity of selected essential oils against Staphylococcus spp. isolated from human semen. Antibiotics.

[40] Sharafi M., Borghei-Rad S.M., Hezavehei M., Shahverdi A., Benson J.D. (2022). Cryopreservation of semen in domestic animals: A review of current challenges, applications, and prospective strategies. Animals.

[41] Leão A.P.A., de Souza A.V., Mesquita N.F., Pereira L.J., Zangeronimo M.G. (2021). Antioxidant enrichment of rooster semen extenders–a systematic review. Res. Vet. Sci..

[42] du Plessis S.S., Agarwal A., Mohanty G., Van der Linde M. (2015). Oxidative phosphorylation versus glycolysis: What fuel do spermatozoa use?. Asian J. Androl..

[43] Taskin A., Ergun F., Karadavut U., Ergun D. (2022). Effect of different extenders on sperm motility and vitality in goose semen cryopreservation. Braz. J. Poult. Sci..

[44] Sukanya R., Phubet S., Srisuwan C., Visid T. (2018). Effects of sugar types in semen extender on sperm quality and longevity of frozen goat semen. Int. J. Vet. Sci..

[45] El-Sheshtawy R.I., Sisy G.A., El-Nattat W.S. (2015). Effects of different concentrations of sucrose or trehalose on the post-thawing quality of cattle bull semen. Asian Pac. J. Reprod..

[46] Getachew T., Goshu G., Lemma A. (2023). Effects of Using Commercial and Homemade Extenders on Sperm Quality of Liquid Stored Semen of Horro Chicken Breed. J. World Poult. Res..

[47] Arıcı R., Günay E., Şenlikci H., Yağcıoğlu S., Eser A., Sandal A., Demir K., Alkan S. (2025). Evaluation of the efficacy of different semen extenders for chilled storage of Aseel rooster sperm. Pol. J. Vet. Sci..

[48] Łukaszewicz E., Jerysz A., Chełmońska B. (2020). Effect of semen extenders and storage time on quality of Muscovy duck (*Cairina moschata*) drake semen during the entire reproductive season. Reprod. Domest. Anim..

[49] Telnoni S., Arifiantini R.I., Yusuf T., Darwati S. (2017). SK Kedu semen cryopreservation in Beltsville poultry semen extender and lactated ringer’s-egg yolk extender using dimethyl sulfoxide. Asian J. Poult. Sci..

[50] Snoeck P.P.d.N., Pessoa T.H.O., Pereira M.G.S., Bastos I.C.L., Melo M.I.V.d. (2019). Can we use LDL instead of egg yolk in BotuCrio^®^ extender to cryopreserve sperm from the Mangalarga Marchador stallion?. Anim. Reprod..

[51] Álvarez C., Luño V., González N., Guerra P., Gil L. (2019). Effect of mare colostrum in extenders for freezing stallion semen. J. Equine Vet. Sci..

[52] Boni R., Ruggiero R., Di Palma T., Ferrara M.A., Preziosi G., Cecchini Gualandi S. (2024). Stallion Sperm Freezing with Different Extenders: Role of Antioxidant Activity and Nitric Oxide Production. Animals.

[53] Thema M.A., Mphaphathi M.L., Ledwaba M.R., Nedambale T.L. (2022). Investigation of the efficacy of different semen extenders and in vitro storage period on Windsnyer boar sperm quality equilibrated at 18 °C. Reprod. Domest. Anim..

[54] Thema M., Mphaphathi M., Ledwaba M., Nedambale T. (2022). Comparison of different extenders and storage temperature on sperm quality of Windsnyer boar semen. Reprod. Fertil. Dev..

[55] Gao D., Zhou X. (2012). Prevention of lethal osmotic injury to cells during addition and removal of cryoprotective agents: Theory and technology. Current Frontiers in Cryobiology.

[56] Tai J.-J.L., Chen J., Wu K., Wang S., Tai C. (2001). Cryopreservation of gander semen. Br. Poult. Sci..

[57] Santiago-Moreno J., Toledano-Díaz A., Castaño C., Velázquez R., Bóveda P., O’Brien E., Peris-Frau P., Pequeño B., Martínez-Madrid B., Esteso M. (2023). Sperm cryopreservation in wild small ruminants: Morphometric, endocrine and molecular basis of cryoresistance. Animal.

[58] antiago-Moreno J., Castaño C., Toledano-Díaz A., Coloma M., López-Sebastián A., Prieto M., Campo J. (2011). Semen cryopreservation for the creation of a Spanish poultry breeds cryobank: Optimization of freezing rate and equilibration time. Poult. Sci..

[59] Tselutin K., Narubina L., Mavrodina T., Tur B. (1995). Cryopreservation of poultry semen. Br. Poult. Sci..

[60] Mussa N., Ratchamak R., Ratsiri T., Kheawkanha T., Vongpralub T., Boonkum W., Chantikisakul V. (2020). Preliminary study on the sperm fatty acid profiles of native and commercial chicken breeds. Proceedings of the 2nd International Conference on Tropical Animal Science and Production (TASP 2019) and the 2nd International Conference on Native Chicken (ICONC 2019), Nakhon Ratchasima, Thailand, 10–12 July 2019.

[61] Iaffaldano N., Romagnoli L., Manchisi A., Rosato M.P. (2011). Cryopreservation of turkey semen by the pellet method: Effects of variables such as the extender, cryoprotectant concentration, cooling time and warming temperature on sperm quality determined through principal components analysis. Theriogenology.

[62] Kuželová L., Vašíček J., Marko Jr H., Marko H., Chrenek P. (2023). Optimization of protocol for stallion semen cryopreservation. Danub. Anim. Genet. Resour..

[63] Pardyak L., Liszewska E., Judycka S., Machcińska-Zielińska S., Karol H., Dietrich M.A., Gojło E., Arent Z., Bilińska B., Rusco G. (2025). The effect of cryopreservation on the turkey (*Meleagris gallopavo*) semen proteome. Theriogenology.

[64] Martín A., Castaño C., O’Brien E., Toledano-Díaz A., Guerra R., Gómez-Guillamón F., Santiago-Moreno J. (2023). Equilibration time improves the sperm variables of wild ruminant ejaculated and epididymal sperm cryopreserved by ultra-rapid freezing. Cryobiology.

[65] Santos C.S., Silva A.R. (2020). Current and alternative trends in antibacterial agents used in mammalian semen technology. Anim. Reprod..

